# Utility of Iron Staining in Identifying the Cause of Renal Allograft Dysfunction in Patients with Sickle Cell Disease 

**DOI:** 10.1155/2015/528792

**Published:** 2015-12-01

**Authors:** Yingchun Wang, Mona Doshi, Salman Khan, Wei Li, Ping L. Zhang

**Affiliations:** ^1^Department of Anatomic and Clinical Pathology, Beaumont Health System, Royal Oak, MI 48073, USA; ^2^Division of Nephrology, Wayne State University School of Medicine, Detroit, MI 48201, USA; ^3^Dallas Nephrology Associates, Dallas, TX 75204, USA

## Abstract

Sickle cell nephropathy (SCN) is associated with iron/heme deposition in proximal renal tubules and related acute tubular injury (ATI). Here we report the utility of iron staining in differentiating causes of renal allograft dysfunction in patients with a history of sickle cell disease. Case 1: the patient developed acute allograft dysfunction two years after renal transplant. Her renal biopsy showed ATI, supported by patchy loss of brush border and positive staining of kidney injury molecule-1 in proximal tubular epithelial cells, where diffuse increase in iron staining (2+) was present. This indicated that ATI likely resulted from iron/heme toxicity to proximal tubules. Electron microscope confirmed aggregated sickle RBCs in glomeruli, indicating a recurrent SCN. Case 2: four years after renal transplant, the patient developed acute allograft dysfunction and became positive for serum donor-specific antibody. His renal biopsy revealed thrombotic microangiopathy (TMA) and diffuse positive C4d stain in peritubular capillaries. Iron staining was negative in the renal tubules, implying that TMA was likely associated with acute antibody-mediated rejection (AAMR, type 2) rather than recurrent SCN. These case reports imply that iron staining is an inexpensive but effective method in distinguishing SCN-associated renal injury in allograft kidney from other etiologies.

## 1. Introduction

In native renal biopsies from patients with sickle cell disease (SCD), sickle cell nephropathy (SCN) may include dominant glomerular disorders, renal tubular injury, or both. SCN has been considered predominantly a vascular disorder with sickle shaped red blood cells (RBCs) clogging the peritubular capillaries leading to papillary necrosis and scarring. Membranoproliferative changes similar to chronic thrombotic microangiopathy (TMA) are the common findings [1–3], as clustered sickle cells result in thrombotic injury. In addition to nonspecific tubular atrophy, due to hemolysis of the vulnerable sickled RBCs, iron/heme deposition in the renal tubules has been described as a tubular injury pattern causing renal failure in native kidneys [4–6]. However, not all renal disease in patients with SCD is due to SCN. Further, there is no pathognomonic lesion that defines SCN. Currently, there is no reliable test to distinguish SCN-associated renal tubular injury from other injury etiologies.

Acute tubular injury (ATI) is also a common finding in renal transplant biopsies, up to 28% of all renal transplant biopsies in our institution (unpublished observation). The most common cause of ATI results from ischemic-reperfusion injury occurring shortly after deceased renal grafts. Calcineurin toxicity, volume depletion, and acute antibody-mediated rejection are other possible causes.

Here we present how to use iron staining to differentiate SCN-associated tubular injury from other etiologies in two renal transplant biopsies from patients with known SCD.

## 2. Cases Presentation

### 2.1. Case  1

A 46-year-old woman with a medical history of SCD and end stage renal disease (ESRD) underwent renal transplantation two years before this biopsy. She was found to have elevated serum creatinine up to 1.4 mg/dL from baseline of 0.9 mg/dL, and her renal graft was biopsied to rule out rejection.

**Figure 1:**
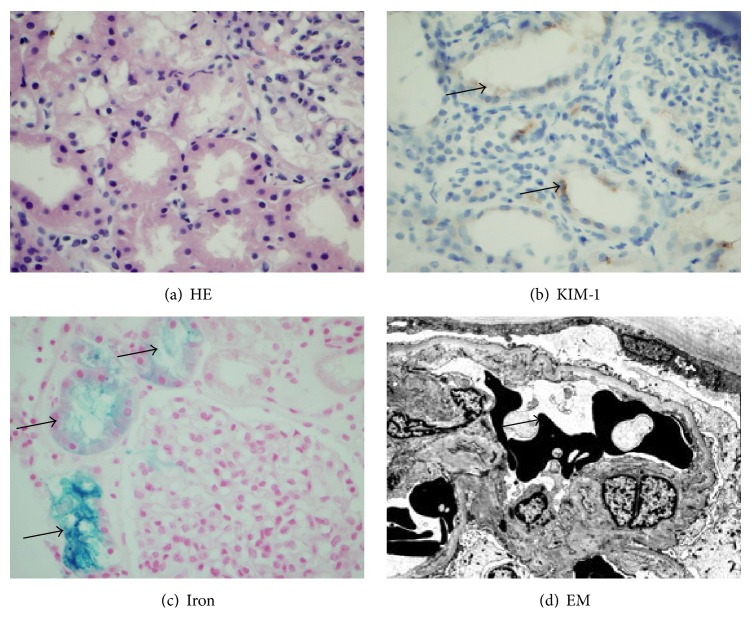
Iron deposition associated with acute tubular injury in the allograft kidney. (a) Dilated proximal tubules with focal disruption of brush border. No inflammatory infiltration is seen (hematoxylin and eosin, original magnification ×400). (b) Acute tubular injury was confirmed by positive kidney injury molecule-1 staining (arrow) along the luminal surface of proximal tubules (original magnification ×400). (c) Iron staining was moderately (2+) positive in the injured proximal tubules (arrow) (original magnification ×400). (d) Electron photomicrograph depicting sickle shaped red blood cells (arrow) clustered in the glomerular capillary loops (original magnification ×4000).

The hematoxylin and eosin (HE) sections revealed three biopsy cores with 10 glomeruli. None of the glomeruli was either segmentally or globally sclerosed. There was no glomerulitis or double contours of glomerular basement membranes noted. Except for focal tubular atrophy, the majority of renal tubules were back-to-back without significant interstitial fibrosis (Figure 1(a)). The proximal tubules were mildly dilated with focal disruption of the brush borders. There was no significant inflammatory infiltration in the renal parenchyma, tubulitis, or vasculitis. BK virus staining was negative with a good positive control. C4d staining by immunofluorescent method was negative in peritubular capillaries. There was no specific glomerular staining for IgA, IgG, IgM, C1q, C3, kappa, or lambda light chain. An immunohistochemical stain for kidney injury molecule-1 (KIM-1) was positive along the luminal surface of some proximal tubules (Figure 1(b)), consistent with acute tubular injury. Approximately 20% of proximal tubules stained 1+ to 2+ positive for iron (Figure 1(c)), implying that the ATI was associated with iron/heme deposition from hemolyzed sickled RBCs into the renal tubules. Two glomeruli were present on Methylene Blue Azure II stained sections. Ultrastructurally, there was good preservation of foot processes. Focal thickening of glomerular basement membranes was noted. Some irregular shaped RBCs were identified in the glomerular capillary loops, consistent with sickled RBCs (Figure 1(d)). No immune complex deposits were present in the mesangial areas, subendothelial or subepithelial spaces. In summary, the mild ATI was most likely associated with iron nephrotoxicity secondary to recurrent SCN.

### 2.2. Case  2

The 35-year-old man underwent a renal transplantation four years before due to SCN-associated ESRD. His recent serum test was positive for donor-specific antibody, and his current serum creatinine was elevated to 3.8 mg/dL before the renal allograft biopsy.

**Figure 2:**
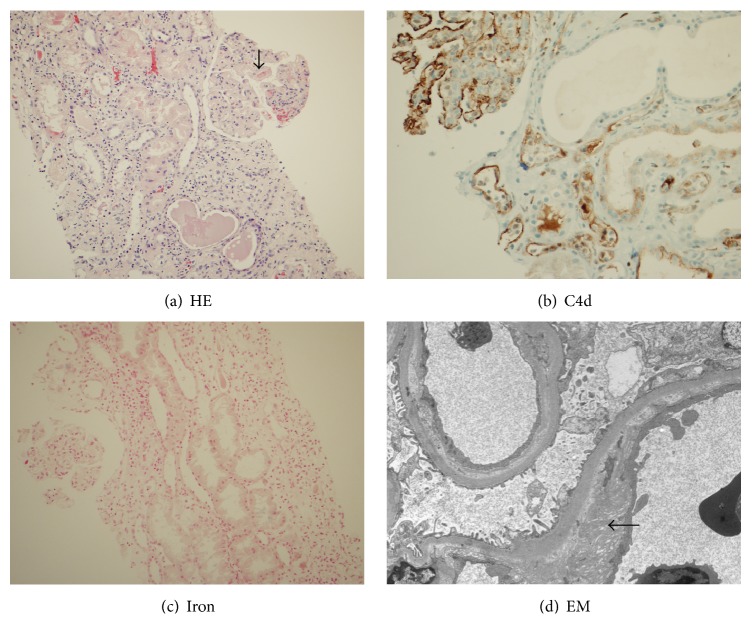
Acute tubular injury caused by type 2 antibody-mediated rejection in the allograft kidney. (a) Light microscopy showed a glomerulus with lobular configuration and focal fibrin thrombi (arrow). Renal tubules are mildly dilated with mild tubulitis. Interstitial mononuclear infiltrate is also seen (hematoxylin and eosin, original magnification ×100). (b) C4d diffusely positive in peritubular capillaries and glomerular capillary loops (original magnification ×200). (c) Iron staining is essentially negative (original magnification ×100). (d) Electron photomicrograph showed double contoured glomerular capillary loops with lucency and subendothelial deposit of fibrin (arrow), consistent with thrombotic microangiopathy (original magnification ×4000).

Sections of light microscopy revealed two biopsy cores of renal cortex containing totally ten glomeruli (Figure 2(a)). One glomerulus was globally sclerosed and the remaining glomeruli showed membranoproliferative pattern with thrombi in one glomerulus. There was lymphocytic infiltration involving more than 20% of the renal cortex with mild tubulitis. No vasculitis was identified. Trichrome-stained section showed mild to moderate interstitial fibrosis and tubular atrophy. BK virus staining was negative with a good positive control. C4d staining by immunohistochemical method was strongly and diffusely positive in glomeruli and peritubular capillaries (Figure 2(b)). Iron staining was essentially negative in the renal tubules (Figure 2(c)). Three glomeruli were present for immunofluorescence studies. At least two glomeruli showed segmentally positive staining for fibrinogen (2+) and IgM (1+). The remaining staining including IgG, IgA, C3, C1q, kappa, and lambda was negative. C4d stained sections showed strong positive staining in 80% of the peritubular capillaries, consistent with antibody-mediated rejection. Four glomeruli were identified on Methylene Blue Azure II stained sections for electron microscopy. Ultrastructurally, there was segmental fusion of foot processes. The glomerular basement membrane showed prominent double contour with lucent appearance and scattered foci of subendothelial fibrin deposits (Figure 2(d)). Occasional irregular shaped RBCs were present, but they do not form thrombus. No immune complex deposits were identified in the mesangial areas, subendothelial or subepithelial spaces. We commented that the TMA can be either secondary to the antibody-mediated rejection or SCN. However, the negative iron stain in the renal tubules implied that SCN was not the major cause for this current TMA. Instead, the TMA was most likely associated with the antibody-mediated rejection and thus classified as type II acute antibody-mediated rejection.

### 2.3. Prussian Blue Iron Staining

The intensity for Prussian blue iron staining within the luminal surface of proximal tubular epithelial cells was graded from 0 to 3+ (0, no staining; 1+, weak fine granular staining along the luminal surface; 2+, moderate granular staining; and 3+, strong large granular staining).

### 2.4. Immunohistochemistry

Immunohistochemistry on renal biopsy was performed as previously described [7]. KIM-1, AKG7 monoclonal antibody, was kindly provided by Dr. Joseph V. Bonventre, Renal Division, Brigham and Women's Hospital, Boston. C4d, rabbit polyclonal antibody, was from Cell Marque, Rocklin, California.

## 3. Discussion

**Figure 3:**
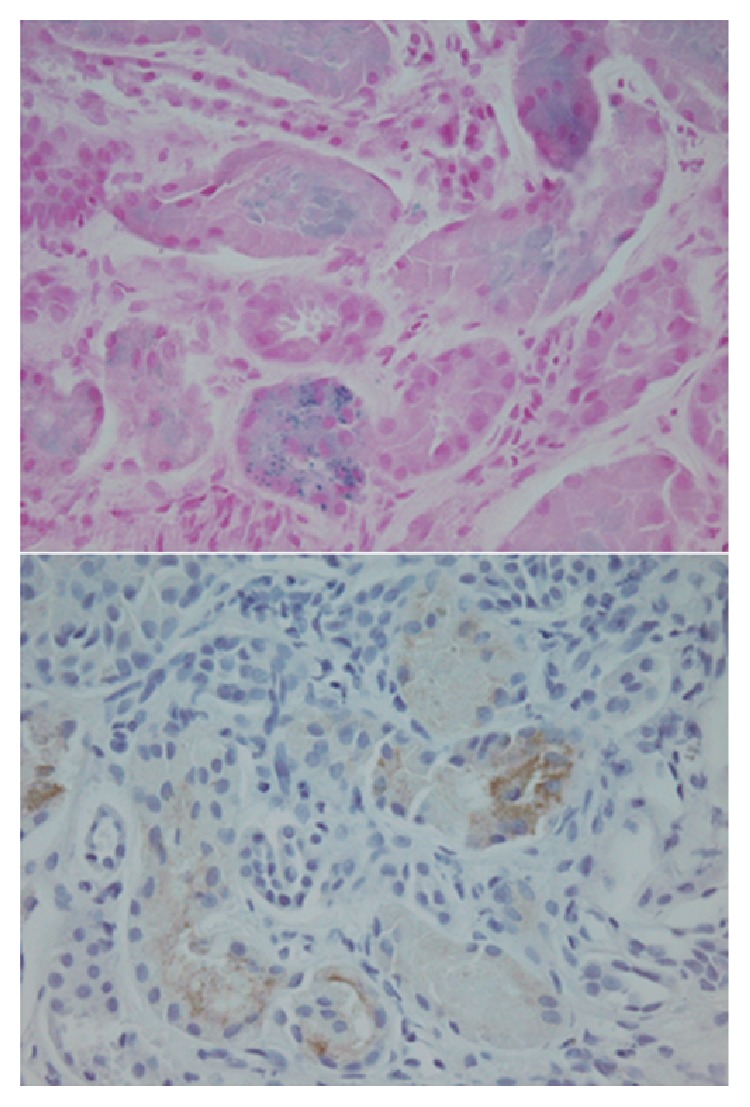
Native renal biopsy with sickle cell nephropathy associated iron deposition and acute tubular injury. A 17-year-old male, with a medical history of SCD, developed acute renal failure. Findings of his renal biopsy were consistent with SCN. Top panel: the proximal tubules were deposited with large amount of iron broken from sickle red blood cells (positive Prussian blue iron staining). Bottom panel: the iron-associated nephrotoxicity in the proximal tubules was confirmed by positive KIM-1 staining.* (Top and bottom panels, original magnification ×400.)*

SCD in native kidneys can be associated with iron/heme deposition in renal tubules and its related acute tubular injury (Figure 3), as reported previously [4–6]. In order to evaluate the utility of iron staining in renal tubules to identify SCN-associated tubular injury from other causes, we also performed Prussian blue iron staining in 29 renal biopsies without a history of SCD. These included 25 biopsies from native kidneys, 13 TMA and 12 IgA nephropathy, and four ATI, one acute cellular rejection and one acute antibody-mediated rejection from allograft kidneys. Among the 13 TMA cases, three had 1+ weak iron staining in less than 10% of renal tubules, whereas the remaining ten cases stained negative for iron. Among the 12 cases of IgA nephropathy, three cases showed 1+ iron staining in less than 1% of the renal tubules, while the other nine cases were negatively stained for tubular iron. In allograft kidneys, four cases with ATI and one case with acute cellular rejection stained negatively for the tubular iron, and one case with antibody-mediated rejection was found to have 1+ iron stain in less than 1% of renal tubules. In the IgA nephropathy and TMA, normal shaped RBCs that went through nephrons are unlikely to be damaged, and thus, there was minimal iron spill into renal tubules. However, renal deposits of iron, hemosiderosis, have been observed in a number of diseases featuring intravascular hemolysis [8], including SCD. Mechanical trauma to erythrocytes liberates hemoglobin into plasma, where hemoglobin is bound by haptoglobin and degraded or filtered and incorporated into proximal tubules through the megalin-cubilin receptor system present on the apical surface of these cells. Heme proteins can cause acute kidney injury through three main mechanisms: decreased renal perfusion, direct cytotoxicity, and intratubular casts formed from the interaction of heme proteins with Tamm-Horsfall protein. Cytotoxic effects of large amounts of heme result from its lipophilic, oxidant, proinflammatory, and apoptotic effects [9, 10].

In our current transplant Case  1, the strong iron staining was found in allograft renal tubules, most likely being the cause of ATI, which was confirmed by positive staining for kidney injury molecule-1, a specific injury marker of proximal tubules [11, 12]. In the second renal transplant case, iron staining was essentially negative in renal tubules, while a type 2 acute antibody-mediated rejection was identified in the allograft biopsy, which is known to cause ischemia type of insult to renal glomeruli and tubules [13, 14]. The different iron staining results from these renal allograft biopsies indicate that the inexpensive iron staining could help differentiate allograft dysfunction secondary to recurrent SCN from other etiologies in patients with known SCD.
